# Zinc Mono-Therapy in Pre-Symptomatic Chinese Children with Wilson Disease: A Single Center, Retrospective Study

**DOI:** 10.1371/journal.pone.0086168

**Published:** 2014-01-24

**Authors:** Kuerbanjiang Abuduxikuer, Jian-She Wang

**Affiliations:** 1 Liver Center, Children's Hospital of Fudan University, Shanghai, China; 2 Department of Pediatrics, Jinshan Hospital of Fudan University, Shanghai, China; Nottingham University, United Kingdom

## Abstract

**Background:**

There is no official consensus regarding zinc therapy in pre-symptomatic children with Wilson Disease (WD); more data is needed.

**Objective:**

To investigate the safety and efficacy of zinc gluconate therapy for Chinese children with pre-symptomatic WD.

**Methods:**

We retrospectively analyzed pre-symptomatic children receiving zinc gluconate in a single Chinese center specialized in pediatric hepatology. Short-term follow-up data on safety and efficacy were presented, and effects of different zinc dosages were compared.

**Results:**

30 children (21 males) aged 2.7 to 16.8 years were followed for up to 4.4 years; 26 (87%) children had abnormal ALT at baseline. Most patients (73%) received higher than the currently recommended dose of elemental zinc. Zinc gluconate significantly reduced mean ALT (p<0.0001), AST (p<0.0001), GGT (p<0.0001) levels after 1 month, and urinary copper excretion after 6 months (p<0.0054). Mean direct bilirubin levels dropped significantly at 1 month (p = 0.0175), 3 months (p = 0.0010), and 6 months (p = 0.0036). Serum zinc levels gradually increased and reached a significantly higher level after 6 months (p<0.0026), reflecting good compliance with the therapy. Complete blood count parameters did not change throughout the analysis period. 8 children experienced mild and transient gastrointestinal side effects. The higher zinc dose did not affect treatment response and was not associated with different or increased side effects when compared to conventional zinc dose.

**Conclusion:**

In our cohort, zinc gluconate therapy for Chinese children with pre-symptomatic WD was effective, and higher initial dose of elemental zinc had the same level of efficacy as the conventional dose.

## Introduction

Wilson disease (WD) is an inborn error of copper metabolism characterized by impaired hepatocellular utilization and biliary excretion of copper. This leads to copper accumulation in the liver, brain, cornea, kidney, and other organs, eventually causing end stage liver disease and severe brain damage [1]. It is caused by autosomal recessive mutations in the *ATP7B* gene [2]. WD has worldwide incidence of 1/35,000–1/100,000 [3], but the exact incidence in China is not published.

A diagnostic scoring system using clinical findings, lab results, liver biopsy, and mutation analysis was developed by the working party of 8^th^ International Meeting on Wilson Disease and Menkes Disease [4], when this was validated in children it had a high sensitivity (98.14%) and specificity (96.59%) [5].

Current treatment regiments include copper-chelators, and zinc salts. The goal of therapy for all patients is to establish, and maintain normal copper homeostasis. This can be achieved with medical therapy or, if rescue treatment fails and in advanced cases, liver transplantation. Treatment of WD can generally prevent, stabilize, or reverse copper overload and symptoms caused by it. Early diagnosis is important, because treatment of the disease is more effective, if initiated at an early stage [6].

The latest European Association for the Study of the Liver (EASL) guidelines [7] recommended zinc for pre-symptomatic patients while acknowledging its role in patients with neurological manifestation. The American Association for Study of Liver Diseases (AASLD) [8] also recommended using zinc for the treatment of pre-symptomatic patients or those on maintenance therapy. Routine follow-up and monitoring of liver function, parameters of copper/zinc metabolism, international normalized ratio, complete blood count, and urinalysis was recommended [9], [10].

A recent systematic review [11] on the treatment of WD found only four qualified studies evaluating zinc mono-therapy [12]–[15], and two studies comparing the efficacy of zinc and penicillamine [16], [17]. Based on limited data, the authors recommended zinc mono-therapy to pre-symptomatic WD patients while waiting for larger, randomized controlled studies. However, both guidelines and the systematic review failed to issue specific recommendation for pediatric patients. More data on treatment is needed for children, especially at pre-symptomatic stage.

With the introduction of gene sequencing technology, an increasing number of Chinese children with WD are being diagnosed before symptoms occur [15]. From 2006, our institution adopted zinc mono-therapy as the first-line treatment for pre-symptomatic children with WD. Here we report a retrospective analysis of 30 pre-symptomatic WD children that received zinc mono-therapy from a single pediatric liver center.

## Clinical Setting, Patients and Methods

### Inclusion/Exclusion Criteria

Children's Hospital of Fudan University is one of the major pediatric tertiary health care centers in China, and mainly receives referrals from the eastern region of the country. Recently, more and more patients all across China are coming to the Liver Center for diagnosis and treatment.

Medical records of children with WD treated on an outpatient basis by one liver specialist (Professor Wang JS) in Children's Hospital of Fudan University from January 2006 to March 2013 were retrospectively analyzed. The Institutional Review Board in Children's Hospital of Fudan University approved this study and waived the need for parental informed consent for children's clinical data to be used for retrospective analysis.

WD Scores were calculated for each child (aged less than 18 years at the time of first visit) using serum ceruloplasmin levels, urinary copper levels, presence/absence of Kayser-Fleischer (K-F) ring, and mutation analysis of *ATP7B* gene [4]. WD Scores of 4 or above were considered diagnostic of WD. *ATP7B* gene mutation analysis was done at the time of diagnosis by Sanger sequencing of DNA extracted from peripheral white blood cells.

Patients were regarded as pre-symptomatic if diagnosed after accidental findings of abnormal liver enzymes, or by family screening after an index patient was confirmed. Children included in this analysis were newly diagnosed, and had baseline data plus at least one follow-up data point. Neurologic manifestations and other causes of liver, muscular, and metabolic diseases were excluded with appropriate investigations. Patients with insufficient data, neurologic/psychiatric symptoms, liver failure, and those who received chelation or combination therapy prior to zinc mono-therapy were excluded from this study.

### Data Collection and Analysis

We recorded baseline characteristics for all included cases, including age, gender, presence/absence of hepatomegaly, splenomegaly and K-F rings. Liver function test results, serum ceruloplasmin/copper/zinc levels, complete blood count, urinary copper levels, urinary analysis, *ATP7B* gene mutations, and initial zinc dosage were collected at the start of zinc therapy. Available follow-up data were collected at 1 month, 3 months and 6 months after the start of zinc therapy, and every 6 months thereafter. Follow-up clinical and laboratory data were compared to baseline data for analyzing the efficacy, and safety of zinc therapy.

### Statistical Analysis

Statistical analyses were performed with STATA software (version 12.0 Special Edition, STATA Corp, College Station, TX). The Pearson chi-square test was used to compare categorical variables, and Fisher's exact values were calculated when expected frequency was five or less. A two independent samples t-test was used to compare continuous variable. Differences between paired data were calculated using a paired t-test. A two-sided p value of less than 0.05 was considered statistically significant.

## Results

A search of out-patient records from January 2006 to March 2013 revealed 49 patients with WD. We excluded 9 patients (cases 31 through 39) for lack of follow-up data either because they were lost to follow-up, or transferred to local care. 5 cases (cases 40–44) were older than 18 years at first visit and were not included in the analysis. Three children (cases 45–47) were excluded for previously receiving chelation/combination therapy, one (case 48) for having epilepsy and hand tremor plus chelation therapy, and one (case 49) for having liver de-compensation at presentation. This patient with liver de-compensation had marked jaundice (total bilirubin level: 136 umol/L), liver cirrhosis with severe splenomegaly and thrombocytopenia, but the albumin level was normal. Basic characteristics of all included and excluded cases were summarized in Table 1.

**Table 1:** Basic Characteristics of Included WD Children At the Start of Zinc Therapy (Cases 1–30), and Cases Excluded From the Analysis (Cases 31–49).

Case No	Sex	Age (Year)	Total Follow-up Time (Year)	Wilson Disease Score					ALT (IU/L)	AST (IU/L)	Hepatomegaly	Splenomegaly	Palmar Erythema	Specific Mutations and/or Amino Acid Changes (**Homozygous, *Heterozygous, ∼Parental Origins Confirmed, Bold: Novel Mutation)	Initial Zinc Dose (mg) (Bold: higher than conventional dose)
				Ceruloplasmin	Urinary Copper	K-F Ring	Mutation	Total Score							
1	M	2.7	4.4	2	2	0	NA	4	420	254	**+**	**−**	**+**	NA	**210**
2	M	6.6	3.7	2	2	0	NA	4	221	211	**+**	**−**	**−**	NA	100
3	F	5.9	3.7	2	2	0	NA	4	214	219	**−**	**−**	**+**	NA	100
4	F	4.3	2.8	2	2	0	4	8	207	183	**−**	**−**	**−**	*c.2924C>A, p.Ser975Tyr/*c.3443T>C,p.Ile1148Thr	**90**
5	M	7.3	2.6	2	2	0	1	5	140	154	**−**	**−**	**−**	*c.2333G>T,p.Arg778Leu	**150**
6	M	3.1	2.6	2	2	0	4	8	291	229	**+**	**−**	**−**	*c.2975C>T,p.Pro992Leu/*c.3809A>G,p.Asn1270Ser	**150**
7	M	3.2	2.5	2	1	0	4	7	7	14	**+**	**+**	**−**	∼*c.2333G>T,p.Arg778Leu/∼*c.3316G>A,p.Val1106Ile	**90**
8	M	4.3	2.4	2	2	0	4	8	124	76	**+**	**−**	**+**	**c.2333G>T, p.Arg778Leu	**150**
9	M	5.8	1.8	2	2	0	4	8	241	153	**−**	**−**	**−**	*c.2145C>A,p.Tyr715Stop/*c.2333G>T,p.Arg778Leu	60
10	M	6.9	1.5	2	2	0	4	8	223	118	**+**	**−**	**−**	**c.2304dupC,p.Met769HisfsX26	80
11	F	3.7	1.4	2	2	2	4	10	87	62	**+**	**−**	**−**	*c.2804C>T, p.Thr935Met/*c.2975C>T,p.Pro992Leu	**90**
12	F	3.6	1.3	2	2	0	4	8	289	197	**−**	**−**	**−**	∼**c.2333G>T, p.Arg778Leu	**90**
13	M	4.3	1.3	2	2	0	4	8	254	116	**−**	**−**	**−**	*c.2755C>G, p.Arg919Gly/*c.4013T>G,p.Ile1338Ser	**150**
14	M	3.9	1.4	2	2	0	4	8	163	125	**−**	**−**	**+**	∼*c.2333G>T, p.Arg778Leu/∼*c.2804C>T, p.Thr935Met	**90**
15	F	12.6	1.3	2	2	0	4	8	111	47	**+**	**−**	**−**	∼*c.2333G>T,p.Arg778Leu/∼*c.2828G>A,p.Gly943Asp	90
16	M	11.7	0.8	2	2	0	NA	4	389	175	**−**	**−**	**−**	NA	90
17	M	8.9	1.0	2	2	0	NA	4	93	71	**+**	**−**	**+**	NA	**150**
18	M	9.8	1.0	2	1	0	1	4	54	40	**+**	**−**	**+**	*c.2333G>T,p.Arg778Leu	**150**
19	M	6.1	1.0	2	2	0	4	8	270	144	**+**	**−**	**+**	*c.1543+1G>T/*c.3041C>T,p.Pro1014Leu	**150**
20	F	4.3	0.9	2	2	0	4	8	197	121	**+**	**−**	**−**	**c.3517G>A,p.Glu1173Lys	**150**
21	M	8.5	1.3	2	2	0	NA	4	72	40	**−**	**−**	**−**	NA	**150**
22	M	4.8	0.8	2	2	0	4	8	88	131	**+**	**−**	**+**	*c.2078C>G,p.Ser693Cys/*c.2333G>T,p.Arg778Leu	**150**
23	M	4.8	0.8	2	2	0	4	8	86	63	**−**	**−**	**−**	*c.2333G>T,p.Arg778Leu/*c.2333G>A,p.Arg778Gln	**90**
24	F	6.8	0.7	2	2	0	4	8	124	94	**+**	**−**	**−**	*c.2122-1G>T/*c.3044T>C,p.Leu1015Pro	**150**
25	F	3.5	0.6	2	2	0	4	8	360	213	**+**	**−**	**+**	**c.3446G>A, p.Gly1149Glu	**90**
26	M	8.8	0.6	2	2	0	4	8	163	92	**+**	**−**	**−**	*c.2755C>G,p.Arg919Gly/*c.1153G>T,p.Glu385Unk	**150**
27	M	5.8	0.6	2	2	0	4	8	364	167	**+**	**−**	**−**	*c.2333G>T,p.Arg778Leu/*c.2975C>T,p.Pro992Leu	**150**
28	M	15.7	0.7	2	2	0	4	8	148	75	**−**	**−**	**−**	*c.2333G>T,p.Arg778Leu/*c.2383C>T,p.Leu795Phe	150
29	F	16.8	0.3	2	2	0	4	8	50	31	**−**	**+**	**−**	**c.2906G>A,p.Arg969Gln	90
30	M	3.5	0.4	2	2	0	4	8	106	63	**−**	**−**	**−**	*c.2333G>T,p.Arg778Leu/*c.2145C>A,p.Tyr715Stop	**90**
31	F	10.2	3.7	2	2	0	NA	4	169	145	**−**	**−**	**−**	NA	NA
32	F	7.7	3.6	2	2	0	4	8	53	63	**−**	**+**	**−**	*c.2804C>T, p.Thr935Met/*c.3809A>G, p.Asn1270Ser	60
33	F	2.5	2.8	2	NA	NA	4	6	393	213	**−**	**−**	**−**	*c.2333G>T, p.Arg778Leu/*c.2975C>T, p.Pro992Leu	100
34	F	12.3	1.8	2	2	NA	NA	4	24	38	**+**	**+**	**−**	NA	NA
35	M	NA	1.7	2	2	0	NA	4	88	47	**+**	**−**	**−**	NA	NA
36	M	4.3	0.8	2	2	NA	4	8	36	24	**+**	**−**	**−**	*c.2333G>T, p.Arg778Leu/*c.2621C>T, p.Ala874Va	None
37	F	5.3	0.8	2	2	0	NA	4	8	17	**−**	**−**	**+**	NA	NA
38	M	3.0	0.8	2	2	0	4	8	142	70	**+**	**−**	**−**	*c.4114C>T, p.Gln1372Stop/*c.2621C>T,p.Ala874Va	NA
39	F	3.7	0.6	2	2	0	4	8	202	122	**−**	**−**	**−**	*c.2187G>T, p.Met729Ile/*c.2333G>T,p.Arg778Leu/*c.2975C>T, p.Pro992Leu	NA
40	F	22	NA	2	2	0	0	4	218	98	**+**			Normal	NA
41	F	19.8	1.1	2	0	0	4	6	73	75	**−**	**−**	**−**	*c.2804C>T, p.Thr935Met/*c.2333G>T,p.Arg778Le	150
42	M	21.2	1.3	2	2	0	2	6	63	78	**−**	**−**	**−**	*c.2975C>T, p.Pro992Leu	90
43	M	35.5	0.9	2	2	0	0	4	NA	NA	**NA**	**NA**	**NA**	Normal	None
44	M	40.5	0.9	2	2	NA	2	6	110	144	**NA**	**NA**	**NA**	*c.3660-3664insG, p.D1222GfsX36	None
45	F	5.7	6.6	2	2	0	NA	4	396	143	**+**	**−**	**+**	NA	50
46	F	9.5	1.6	2	2	2	NA	6	52	27	**+**	**−**	**−**	NA	None
47	F	13.7	3.6	2	2	0	NA	4	68	53	**+**	**−**	**−**	NA	None
48	F	13.6	5.4	2	2	2	NA	6	24	22	**−**	**−**	**−**	NA	60
49	M	15.8	0.8	2	2	NA	NA	4	28	28	**−**	**+**	**+**	Normal	NA

NA: Not Available.

Consequently, a total of 30 pre-symptomatic pediatric patients were analyzed, and all children had a Wilson Disease Score of 4, or higher. There were 21 males (70%), the mean age at presentation was 6.6 years old (range 2.7–16.8 years), and mean follow-up time was 1.54 years (range 0.3–4.4 years). 17 (57%) children had hepatomegaly, 9 (30%) had palmar erythema, 2 (7%) had splenomegaly, and 1 (3%) was found to have K-F ring after ophthalmological examination. 26 (87%) patients had abnormal alanine amino transferase (ALT) levels (greater than 2-times of upper normal limit) at presentation, while 4 others had normal liver function tests.

Sanger sequencing of *ATP7B* established variants believed to be disease causing in 24 patients. 6 had homozygous mutations, 16 had compound heterozygous mutations, and 2 had only one heterozygous mutation. Parental origins of mutations were confirmed in 1 patient with homozygous mutation, and in 3 children with 2 compound heterozygous mutations (Table 1). 5 variants previously not reported in the Wilson Disease Mutation Database (http://www.wilsondisease.med.ualberta.ca) include 1 homozygous (c.3446G>A), and 4 heterozygous (c.1153G>T, c.3041C>T, c.3044T>C, c.4013T>G) missense mutations (lead to amino acid change). All of these variants were predicted to be disease causing by Mutation Taster (www.mutationtaster.org) and are not seen in over 10,000 US alleles (evs.g.s.washington.edu, accessed 24 Oct 2013). Results of mutation analysis for the remaining 6 children were not recorded in their charts.

### Zinc Therapy

A domestically produced zinc gluconate tablet containing 10 mg of elemental zinc each were used for all patients. A different dose was used for different patients according to their age, liver enzyme levels, degree of hepatomegaly/splenomegaly, predicted gastrointestinal tolerance, and parental concerns. The zinc dose was increased in some patients if their laboratory parameters were not responsive to treatment, or an ALT re-elevation occurred after treatment response. In other patients it was reduced if not tolerated, and then slowly increased to the target dose. Our lowest initial dosage was 30 mg of elemental zinc twice daily, while the highest dosage was 70 mg of elemental zinc three times a day. Only a few children received twice a day dosing due to school attendance. Total daily dosages of elemental zinc given initially were also provided in Table 1. In accordance with published recommendations of zinc dosage in children [12], two groups of patients were identified: “conventional zinc dose” group consisted of children older than 5 years of age who received 100 mg or less daily zinc, although a 15.7 year old adolescent received 150 mg/day zinc and was also included in this group; the “higher zinc dose” group consisted of those less than 5 years who received 90 mg/day or more, and 5–10 year olds who received 150 mg/day or more. Twenty two (73%) children received higher zinc dose, while eight (27%) received conventional dose.

### Treatment Outcome

Of 26 (87%) patients with an abnormal ALT levels at baseline, the cumulative number of children with ALT normalization after 1 month, 3 months, and 6 months of zinc therapy were 17, 19, and 19 respectively. This normalization was maintained for the duration of the study data points available. A further five children achieved liver enzyme normalization after 1 year but before 2 years of therapy. The remaining 2 children have not normalized at their one year follow up. Of 4 patients with normal ALT at first presentation, 2 remained normal under zinc therapy, 1 child had recurrent abnormalities, while the remaining one had an abnormal ALT at 1 month and returned to normal at 6 month.

We compared the number of children with normal ALT levels between the conventional zinc dose group and the high zinc dose group at each of the follow-up time points. No statistically significant differences were observed at all follow-up time-points; higher zinc dose achieved the same proportion of children with normal ALT levels as the conventional zinc dose (Table 2).

**Table 2:** Number of Children with Normal and Abnormal ALT, Comparison of Different Zinc Doses at Each Time Point.

	Number of Children (with normal ALT/with abnormal ALT)						
	1st Visit	1 Month	3 Months	6 Month	1 Year	1.5 Years	2 Years
**Conventional Dose**	1/7	6/2	6/1	4/2	4/0	2/1	2/0
**Higher Dose**	3/19	15/7	13/4	8/5	5/4	1/4	2/0
**P value**	1.000	1.000	1.000	1.000	0.228	0.464	na

na: not applicable.

Patients were regarded as having a quick response to zinc therapy if their liver enzymes dropped to normal levels at 6 moths and remained so afterwards, or liver enzymes remained normal if the child initially presented with normal liver function. In total, 21 patients were categorized as having a quick response. On the other hand, patients were regarded as having slow or no response if ALT levels remained high at 6 months or were normal in the first six months but became abnormal at any time after that. 9 children fulfilled the criteria of slow/no response. The treatment outcome was not associated with age, gender, initial ALT/AST (Aspartate Amino Transferase) levels, presence of other abnormal findings (hepatomegaly, splenomegaly, K-F ring, and palmar erythema), or the initial zinc dose (Table 3).

**Table 3:** Treatment outcome and its' Association with Baseline Characteristics.

Treatment Outcome	Mean Age (Year)	Male (n)	ALT (IU/L)	AST (IU/L)	Presence of Hepatomegaly (n)	Presence of Splenomegaly (n)	Presence of Palmar Erythema (n)	Presence of K-F Ring (n)	High Zinc Dose (n)	Mean Elemental Zinc (mg)/Day
**Quick Response (n = 21)**	6.6	14	166	115	12	2	4	1	16	117
**Slow/No Response (n = 9)**	6.6	7	228	140	5	0	5	0	6	130
**P Value**	0.99	0.68	0.16	0.35	1.00	1.00	0.08	1.00	0.67	0.39

**Figure 1:**
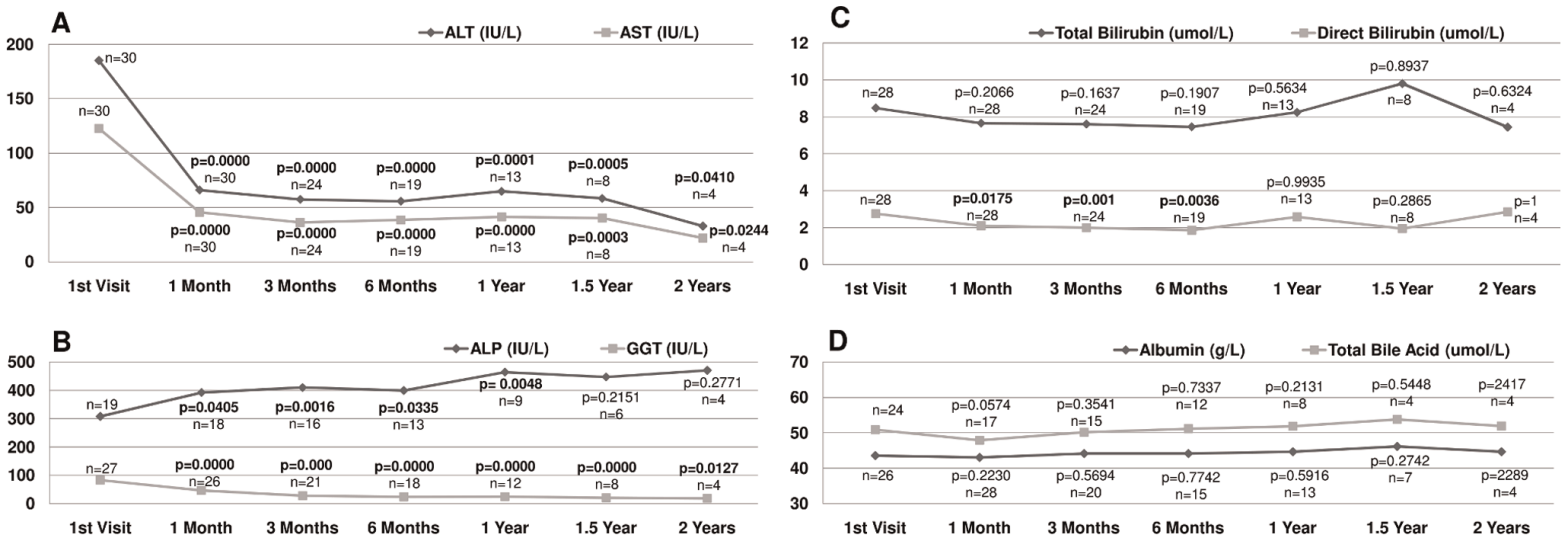
Parameters of Liver function. Linear plot of mean values of liver function parameters at each visit, with number of observations, and p value from paired t-test results with the 1st visit. A: Serum ALT and AST levels. B: Serum ALP and GGT levels. C: Serum total and direct bilirubin levels. D: Serum albumin and total bile acid levels.

**Figure 2:**
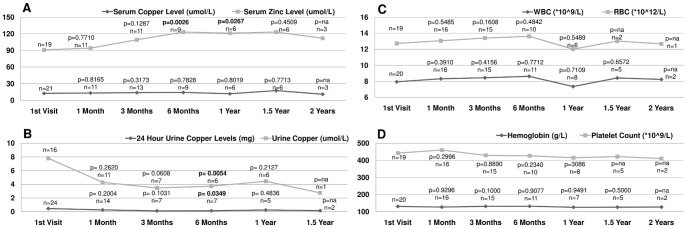
Parameters of copper/zinc metabolism and complete blood count. Linear plot of mean values at each visit with number of observations and p value from paired t-test results with the 1st visit. A: Serum copper and zinc Levels. B: Urinary copper levels. C: White/red blood cell counts. D: Hemoglobin level and platelet counts.

We compared liver function test results, serum copper/zinc levels, urinary copper levels, and complete blood counts at each follow-up to the baseline data using paired t-test. Data after 2 years of follow-up were not analyzed due to insufficient sample size. Results of statistical analysis are provided in Figure 1 and Figure 2.

After 1 month of zinc therapy, the mean serum ALT (p<0.0001), AST (p<0.0001) and Gamma Glutamyl Transpeptidase (GGT) (p<0.0001) levels dropped significantly and remained lower throughout analysis period. However, serum Alkaline Phosphatase (ALP) levels gradually increased after zinc therapy and were significantly higher at 1 month (p = 0.04), 3 months (p = 0.0016), 6 months (p = 0.03), and 1 year (p = 0.0048). Values were not statistically significant beyond 1 year, probably due to a small sample size. There were no statistically significant changes in total bilirubin, serum albumin, and total bile acid levels throughout the follow-up period. Mean direct bilirubin levels remained within normal limits at all time points, but, dropped significantly at 1 month (p = 0.0175), 3 months (p = 0.0010), and 6 months (p = 0.0036), returning to the same level as the baseline at 1 year and thereafter (Figure 1).

Serum copper levels remained constant during the treatment period; however, serum zinc levels gradually increased after zinc therapy with a statistically significant increase at 6 months (p = 0.0026) and 1 year (p = 0.0267), indicating good compliance with zinc therapy. Urine copper concentration and 24-hour urine copper excretion dropped after zinc therapy, but did not reach significantly lower levels until 6 months (p = 0.0054 and 0.0349 respectively). Complete blood count parameters including white blood cell counts, red blood cell counts, hemoglobin levels, and platelet counts did not change after the start of zinc therapy and remained constant until 2 years of follow-up (Figure 2).

### Effect of Different Zinc Doses on Lab Parameters

Lab parameters of patients receiving the conventional zinc dose (n = 8) or high zinc dose (n = 22) were compared at various time points of follow-up. Mean values of the liver function test, copper/zinc metabolism, and complete blood count parameters are shown in Table 4. P values of t-test results are provided for each parameter at each visit. Changes in mean serum ALT, AST, and albumin levels (ALB), white blood cell (WBC), red blood cell (RBC) counts, serum copper (Scu), serum zinc (Szn), urinary copper (Ucu), and 24-hour urine copper levels (24HUcu) were not statistically different when comparing different zinc-dose groups. Mean levels of total bilirubin (TB), direct bilirubin (DB), ALP, and hemoglobulin (Hb) were significantly different at the start of zinc therapy (p = 0.011, 0.003, 0.015, and 0.01 respectively), but these differences disappeared later during the follow-up. AST levels at 2-year follow-up were different according to zinc dose (p = 0.02). At 1-month follow-up, mean platelet (PLT) level was significantly lower in high dose group when compared with conventional dose group (p = 0.0228), but this difference disappeared in later follow-up comparisons. Mean values of WBC, RBC, and Hb levels did not differ with different doses of zinc therapy.

**Table 4:** Effect of Zinc Dose on Liver Function, Copper Metabolism, and Complete Blood Count at Different stages of Follow-up.

Time of Follow-up	Zinc Dose	Liver Function							Copper/Zinc Metabolism				Complete Blood Count			
		ALT	AST	GGT	TB	DB	ALP	ALB	Scu	Szn	Ucu	24HUcu	WBC	RBC	HB	PLT
**Start of Zinc Therapy**	**Normal**	200	129	96	11.2	3.9	216	44	11.5	81	5.5	0.51	6.9	5.1	150	254
	**High**	180	120	79	7.7	2.4	333	43.5	12.9	78	8	0.44	8.1	4.7	129	319
	**P Value**	0.668	0.772	0.367	**0.011**	**0.003**	**0.015**	0.66	0.428	0.615	0.2549	0.7693	0.544	0.164	**0.01**	0.447
**1 Month**	**Normal**	61	43	63	9.9	2.46	416	44.4	14.1	67	3.1	0.27	7.4	4.6	127	440
	**High**	68	47	42	6.8	1.96	386	42.7	12.3	93	4.4	0.27	8.5	4.8	128	309
	**P Value**	0.77	0.705	0.101	**0.038**	0.0803	0.641	0.139	0.422	0.196	0.1202	0.9821	0.436	0.403	0.969	**0.02**
**3 Month**	**Normal**	53	33	24	8.6	2.44	356	44.1	11.8	101	1.7	0.11	8.6	4.9	132	287
	**High**	59	38	30	7.2	1.82	429	44.2	15	91	3.6	0.13	8.4	5	131	305
	**P Value**	0.776	0.594	0.559	0.401	0.1427	0.386	0.967	0.22	0.295	0.085	0.7958	0.886	0.554	0.828	0.558
**6 Month**	**Normal**	51	35	25	9.5	2.25	328	45.7	15.2	122	3.3	0.17	8.9	5.3	139	296
	**High**	58	40	25	6.5	1.68	431	44.3	13.9	105	3.8	0.12	8.5	4.9	128	295
	**P Value**	0.701	0.45	0.939	0.119	0.2478	0.315	0.18	0.716	0.25	0.7691	0.6183	0.815	0.118	0.129	0.976
**1 Year**	**Normal**	39	25	26	8.8	2.25	407	43.9	11.8	101	4.7	0.31	6.9	4.8	127	308
	**High**	76	49	25	8	2.73	481	45	11.8	118	3.7	0.19	7.8	4.5	126	271
	**P Value**	0.251	0.152	0.977	0.835	0.6313	0.443	0.498	0.998	0.306	0.2806	0.2666	0.468	0.58	0.911	0.55
**1.5 Year**	**Normal**	54	31	17	10.4	2.8	445	45	18.8	105	na	0.27	8.1	na	133	319
	**High**	61	46	23	9.8	1.44	449	46.6	17.2	105	na	0.04	8.6	na	122	281
	**P Value**	0.811	0.404	0.57	0.86	0.1604	0.967	0.526	na	na	na	na	0.71	na	0.324	0.582
**2 Year**	**Normal**	35	20	17	11.5	4.4	507	44	10.1	94	na	na	9.2	na	na	na
	**High**	32	25	21	3.4	1.3	435	45.5	12.5	114	na	na	7.3	na	na	na
	**P Value**	0.877	0.02	0.578	0.388	0.2679	0.332	0.36	na	na	na	na	na	na	na	na

na: not applicable due to lack of data to perform t-test. Abbreviations were provided within the text, and units for each parameter were provided in Figure 1 and Figure 2.

### Zinc Toxicity

One 16 year-old girl stopped taking her medicine after experiencing fever and diarrhea several days after initiating zinc therapy at 30 mg 3-times-a-day. She had been identified through family screening, and had normal liver function at presentation. Her liver enzymes remained normal, and she was able to re-start the therapy after one month without any further adverse events. 3 other children experienced mild abdominal discomfort and occasional vomiting, 4 patients had mild abdominal or epi-gastric pain/discomfort. One of these individuals, a 6 year-old girl, was switched to penicillamine, after 3 years of zinc therapy, due to recurrent ALT elevations. The other 3 patients were able to continue zinc therapy despite mild and occasional gastrointestinal problems. Overall, 8 out of 30 (26.7%) children experienced mild gastrointestinal side effects of zinc. Side effects occurred in 3 out of 8 children with conventional zinc dose and 5 out of 22 children with high zinc dose, and occurrence rate wasn't significantly different (p = 0.643).

Zinc treatment did not affect complete blood count parameters throughout the study period. All patients had regular urine analysis. 2 children experienced a mild and transient hematuria (occult blood and red blood cells in urine), no other abnormalities such as persistent hematuria or proteinuria, were observed.

## Discussion

In the 1960s, doctors in Holland started using zinc in a patient with WD and achieved a clinical response in 1.5 years [18]. Later research further illustrated the mechanisms of action [19]–[23]. In previous researches, the dose of elemental zinc for children with WD was determined by age and body weight: 25 mg twice daily if aged 1 to 5 years; 25 mg three time a day if aged 6 to 15 years and body weight is under 125 pounds; 50 mg three times a day if aged 16 years or older [12], [24], [25].

In our single center sample of pre-symptomatic children with WD, most had elevated liver enzymes. It might be reasonable to predict that a higher zinc dose will take less time to achieve treatment goals while producing more side effects. However, our comparison of the number of children with a normal ALT at each time point of follow-up did not reveal any differences between higher zinc dose and conventional zinc dose groups (Table 2). Children in conventional zinc dose group achieved the same rate of quick response as the children in the high zinc dose group (Table 3). No significant dose related difference was observed when comparing the occurrence of gastrointestinal side effects. At different time points of follow-up, the major parameters of liver function, copper/zinc metabolism, and complete blood count did not significantly differ between high and conventional zinc dosage groups with the exception of mean PLT levels at 1 month. However this difference disappeared at subsequent time points (Table 4).

Due to lower body weight and less copper accumulation prior to treatment, Brewer et al. warned against the possibility of over-treating younger children with zinc. They recommended 25 mg of elemental zinc twice daily for children under the age of 5, and 25 mg three times daily for patients aged between 5 and 15 years [12], [25]. Although zinc has an excellent profile with a low incidence of side effects, better efficacy, and low cost, one must be cautious that rare, and severe complications [26]–[29] might occur and regularly check clinical and biochemical parameters, while keeping in mind potential mechanisms of zinc toxicity [30], [31]. In our group, most children received higher than the conventional dose of molecular zinc (22 out of 30). This might explain the reason why more children (8 out of 30, 26.7%) experienced mild gastrointestinal side effects than Brewer et al reported (4 out of 34, 11.76%) [24]. However, our patients with higher zinc dosage did not experience any serious complications, significant increase of side effects, or treatment failure compared to children receiving conventional zinc dose.

Presence of palmar erythema tended to be associated with slow/no response, but the association did not reach statistical significance (p = 0.08). A larger sample size in future studies should be evaluated to see if there is a significant relationship (Table 3).

Yuzbasiyan-Gurkan et al. [32] reported serum ALP changes in WD patients treated with zinc, ALP was elevated above the normal range after a few weeks of zinc therapy and stabilized at the higher end of normal range after 1 year. In our study, mean serum ALP levels gradually increased after zinc therapy, and were significantly higher than baseline at 1 month all the way through the first year, but the statistical significance was lost later (Figure 1). One should also bear in mind that isolated ALP elevation while all other liver function parameters were improving or stable raises the possibility that ALP might originate from bone. Sadighiet al [33] reported that zinc supplementation stimulates bone growth/fracture healing and increases serum ALP level.

Although the mean direct bilirubin level at the first presentation was normal, it dropped significantly after 1 month of zinc treatment, and remained so until 1 year. (Figure 1). Linn [34] administered zinc for 12 WD patients with hepatic manifestation and noted a decrease in bilirubin levels. Other studies with children mentioned serum bilirubin levels remained normal or stable during zinc therapy [24], [35]. In reviewing the literature, we were unable to find previous studies of pre-symptomatic children that document direct bilirubin changes during zinc treatment. It is interesting to note that even a small but significant reduction in mean direct bilirubin levels accompanied improvement of liver enzymes. Our finding of a significant decrease in direct bilirubin levels after zinc therapy in pre-symptomatic WD children needs to be confirmed by future studies, and its significance in evaluating treatment response awaits elucidation.

Brewer reported significant increase in both serum and urinary zinc levels 1 year after zinc mono-therapy in children (p = 0.0001) [24]. The mean serum zinc level in our cohort started to increase after zinc therapy, and was statistically significantly higher than the baseline level at 6 months and 1 year (p = 0.0026 and 0.0267 respectively) (Figure 2). This indicated good compliance among WD patients, as the vast majority of children were receiving the prescribed dose when checked at each visit. The Brewer study compared both plasma and urine copper at baseline to 1 year after zinc mono-therapy in pediatric patients, both parameters dropped significantly (p = 0.022 and 0.0001 respectively) [24]. In our study, both urine copper concentration and 24 urine copper quantification values dropped at the 6-month follow-up (p = 0.03 and 0.005). Conversely, mean serum copper levels failed to change throughout the study period (p = 0.8 at 1 year follow-up) (Figure 2). This lack of detectable change might be related to methodological limitations for measuring serum copper levels in our institution. Specifically, changes in free copper might not be detected due to the fact that majority of serum copper is bound to ceruloplasmin.

### Study Limitations

The main limitation of this study was that it was a retrospective, non-randomised cohort of children treated with different doses of zinc. The study follow up time is significantly shorter than several other studies (mean 1.54 years, range 0.3–4.4 years). However, we have chosen to publish this interim data now to ensure rapid availability to the academic community of our experience with high dose of zinc gluconate therapy. This seems an appropriate time point because we have studied a large enough group of patients to detect any large safety signals and further longitudinal follow up of this cohort is unlikely to lead to further safety signals as the majority of these cases have entered the maintenance therapy phase, where doses are at the same level as previous trials.

We evaluated efficacy based on intention to treat of the initial zinc dose in our cohort of patients. In routine clinical practice, zinc doses were dynamically adjusted according to tolerance and liver enzyme changes. We attempted to collect intermediate and final zinc dose data, but the collected data was inadequate for statistical analysis. A total of 15 patients had available data on intermediate or final zinc dose. Dosages were slightly increased in 10 patients, slightly decreased in 4 patients, and did not change in 1 patient.

We used Fisher's exact test for 2×2 table analysis when comparing small sample sizes. However, some data in Table 2 (statistics at 1 year follow-up), and Table 3 (presence of splenomegaly and K-F ring) might be too small to reflect true differences between groups. In the future, larger studies will be required to evaluate if these differences are truly not significant.

Long-term zinc therapy may interfere with intestinal iron absorption causing anemia in children. Although mean Hb level did not change throughout our study period, we did not routinely test serum iron levels and cannot confirm if there is any sub-clinical iron deficiency that will affect Hb levels with longer zinc therapy.

Adult and adolescent studies showed 24-hour urinary copper and non-ceruloplasmin serum copper as important indicators for evaluating the efficacy of WD treatment [13], [24], [36], [37]. These parameters might not be equally valid in pediatric patients. Mizuochi et al. [35] treated 4 pre-symptomatic children aged 5–7 years with zinc sulfate, and recommended maintaining 24-hour urinary copper excretion between 1 and 3 ug/kg/day might achieve good results in younger children. Observations of 24-hour urine copper per kilogram of body weight and serum non-ceruloplasmin copper levels were not possible in this retrospective study as we did not regularly record body weight and check non-ceruloplasmin copper levels during follow-up visits.

## Conclusion

In our single center experience, zinc gluconate was effective in reducing liver enzymes and urinary copper excretion, while producing few gastrointestinal side effects which were mild and transient. The higher zinc dose achieved the same treatment outcome and caused the same rate of gastrointestinal effect when compared to the currently recommended zinc dose. Based on the data presented in this paper, we have not established a clinically significant benefit in a higher than recommended zinc dose. However, we have also demonstrated that a higher dose of zinc for induction may not be associated with a higher rate of side effects than the conventional dose. Our data suggest that the decrease in serum direct bilirubin level while remaining within the normal range might reflect improvement of liver function, and could be considered as a marker for treatment response.

## Supporting Information

Table S1 **Table S1 provided full follow-up data for each patient at each visit.** Abbreviations were provided within the text.
(XLSX)
