# Correction: Zinc Mono-Therapy in Pre-Symptomatic Chinese Children with Wilson Disease: A Single Center, Retrospective Study

**DOI:** 10.1371/journal.pone.0092491

**Published:** 2014-03-10

**Authors:** The PLOS ONE Staff

Notice of Republication

This article was republished on January 31, 2014, to remove confidential or copyrighted information. Please download the article and supporting information again to view the corrected version.

## References

[1] Abuduxikuer K, Wang J-S (2014) Zinc Mono-Therapy in Pre-Symptomatic Chinese Children with Wilson Disease: A Single Center, Retrospective Study. PLoS ONE 9(1): e86168 doi:10.1371/journal.pone.0086168 24475083 10.1371/journal.pone.0086168 PMC3901685

